# Clinical effects of slim-diet, with lifestyle modification for childhood obesity in community-based healthcare program: A case series

**DOI:** 10.1097/MD.0000000000020817

**Published:** 2020-06-26

**Authors:** Koh-Woon Kim, So-Jin Kim, Hojun Kim, Seung-Uoo Shin, Jaechul Song, Won-Seok Chung

**Affiliations:** a Department of Korean Rehabilitation Medicine, College of Korean Medicine, Kyung Hee University, Seoul; b Department of Health Promotion, Public Health Center, Yongin-si, Gyeonggi-do, Republic of Korea; c Department of Korean Rehabilitation Medicine, College of Korean Medicine, Dongguk University, Goyang-si, Gyeonggi-do; d Shinkang Korean Medicine Clinic; e Symphony Korean Medicine Clinic, Seoul, Republic of Korea.

**Keywords:** childhood obesity, herbal medicine, community care, lifestyle modification

## Abstract

**Rationale:**

Although there are several reports on the effect of herbal medicine on weight loss in adults, evidence supporting its efficacy and safety in obese pediatrics is insufficient. Herein, we clinically investigated the preliminary experience of community-based healthcare program in cases of childhood obesity treated with an herbal complex, Slim-diet (SD), along with lifestyle modification.

**Patient concerns:**

Seventeen subjects with childhood obesity participated in a community-based healthcare program, which consisted of twice-a-week play type physical activity and dietary counseling program with simultaneous twice-a-day administration of SD for 4 weeks.

**Diagnoses:**

The data of 13 obese pediatrics (body mass index [BMI] ≥ the 95th percentile for children of the same age and sex) in their 3rd to 6th grade who finally completed at least 6 visits out of a total of 8 visits of the program including baseline and endpoint assessments were analyzed.

**Interventions:**

Participants received 20 g of SD daily. Simultaneously, play-type physical activity program with an exercise therapist and dietary counseling with a dietitian for lifestyle modification were conducted at every visit. Body composition, blood chemistry, the Korean Youth Physical Activity Questionnaire (KYPAQ) score, and the preference for salt density and sugar content were assessed at baseline and endpoint.

**Outcomes:**

After SD administration, body mass index decreased from 26.74 ± 2.11 kg/m^2^ to 26.50 ± 2.20 kg/m^2^ (*P* < .05) with statistically significant increases in height, weight, and skeletal muscle mass. The results of blood chemistry and the KYPAQ score showed no significant change. The preferences for salt density were improved in 8, maintained in 2, and worsened in 3 participants and those for sugar content were improved in 6 and maintained in 7 participants with no worsening.

**Lessons:**

In the present study, we showed the clinical effects of SD with lifestyle modification in patients with childhood obesity who participated in community-based healthcare program. Further clinical studies investigating the effects of SD are required.

## 1 Introduction

Childhood obesity is a common disease with high risk for multiple comorbidities previously considered to be “adult” diseases, affecting children's health and psychological well-being.^[1–3]^ The prevalence of childhood obesity has increased worldwide over a short period of time, becoming a global epidemic.^[4,5]^ Evidence-based clinical guidelines conclude that treatment programs should be multicomponent, targeting changes in diet, physical activity, and sedentary behaviors.^[6,7]^ Although lifestyle modifications including dietary changes aimed at decreasing caloric intake and increasing the physical activity have shown only moderate effects on weight loss, information on the efficacy and safety of medications and bariatric surgery for weight loss in children is still limited.^[1,8,9]^

Considering the limitations of conventional treatment options for childhood obesity, herbal medicines, which have recently been administered as anti-obesity agents based on the results of several experimental and clinical studies, could be considered complementary and alternative treatment options.^[10–14]^ Recently, clinicians and investigators have significantly emphasized the importance of the socio-environmental context where decisions related to health behaviors are being made and the components of community-based obesity interventions for weight loss in childhood obesity are applied.^[15]^ Still, there is an inconsistency on the effect of multicomponent community-wide interventions in the available studies,^[16]^ and a few clinical trials have examined the effect of community-based lifestyle modification program combined with herbal medicinal treatment for childhood obesity.^[17]^

Herein, we describe the preliminary experience of community-based healthcare program in 13 cases of childhood obesity treated with an herbal complex, Slim-diet (SD), with lifestyle modification. This retrospective study aimed to investigate the effect of community-based lifestyle modification program combined with herbal medicinal treatment on body composition, blood chemistry, physical activities, and preference for salt density and sugar content in obese pediatrics.

## 2 Case report

### 2.1 Patient eligibility

The data of obese pediatrics (body mass index [BMI] ≥ the 95th percentile for children of the same age and sex) in their 3rd to 6th grade who participated in the study and completed at least 6 visits out of a total of 8 visits (twice a week for 4 weeks) of the health promotion service program of Suji-gu Public Healthcare Center (Yongin-si, Gyeonggi-do, Korea) including baseline and endpoint assessments were analyzed. We excluded participants with hereditary disorders or endocrine diseases that could be one of the reasons of symptomatic or secondary obesity. Participants whose guardians voluntarily agreed to participate in the healthcare program and signed written informed consent form including agreement on personal information were finally included in the study. The research protocol was registered with the Clinical Research Information Service of Korea (KCT0003964) and was reviewed and approved by the Institutional Review Board (IRB) of the Korean Medicine Hospital of Kyung Hee University at Gangdong (KHNMCOH 2019-04-002).

### 2.2 Methods and intervention

Seventeen participants participated in community-based healthcare program, which consisted of play-type physical activity program, dietary counseling, and administration of SD, with twice-a-week visits for 4 weeks from July 31 to August 23, 2018. Participants received 20 g of SD (Saerom Pharmaceutical Co., Ltd., Anseong-si, Gyeonggi-do, Korea) daily. The soft extract of SD was obtained by performing the following process: 3 hours of hot water extraction of 15 species of medicinal herbs, which were blended in a specific ratio (Table 1) and concentration and were finally mixed with the concentrate with Oryzae Gluten at a ratio of 4:6. SD was administered twice daily after breakfast and dinner for 4 weeks. Adherence and response to the prescription of SD was monitored by a doctor of Korean medicine licensed by the Korean Ministry of Health and Welfare with greater than 10 years of clinical experience. Simultaneously, play-type physical activity program with an exercise therapist and dietary counseling with a dietitian for lifestyle modification were conducted at every visit.

**Table 1:**
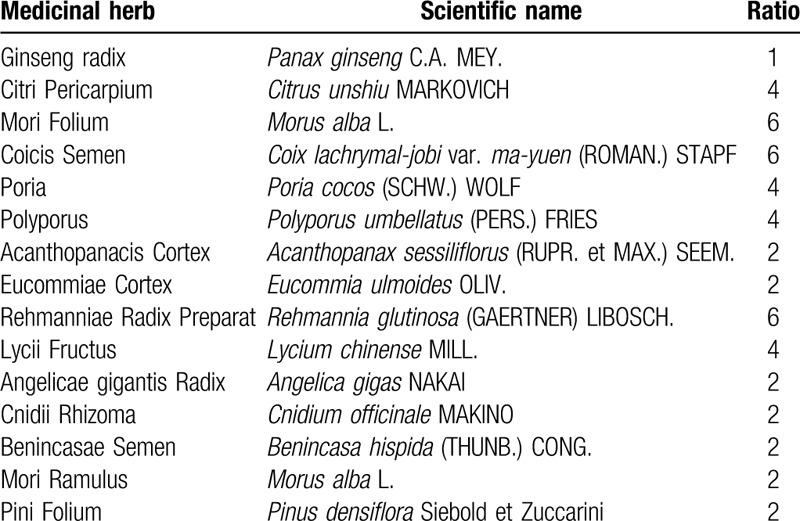
The ratio of medicinal herbs in Slim-diet.

To evaluate the effects of community-based healthcare program with SD, body composition (i.e., BMI, body fat mass [BFM], skeletal muscle mass [SMM], percent body fat) measured using InBody 520 (Biospace, Seoul, Korea), blood chemistry (i.e., fasting blood sugar, total cholesterol, high-density lipoprotein cholesterol, low-density lipoprotein cholesterol [LDL-chol], triglyceride [TG]), the Korean Youth Physical Activity Questionnaire (KYPAQ) score,^[18]^ and 4-step preference for salt density (no salty, less salty, normal, and much salty) and sugar content (no sweet, less sweet, normal, and much sweet) were assessed at baseline and endpoint. Specific symptoms or adverse events were reported at every visit.

### 2.3 Data analysis

Data were presented as mean ± standard deviation. Paired *t* tests and Wilcoxon signed rank tests were conducted to evaluate the mean differences between before and after treatment data. Statistical significant level was *P* < .05. Statistical analysis was performed using Statistical Package for the Social Sciences for Windows (version 18.0; International Business Machines Corp., Armonk, NY).

### 2.4 Results of therapy

Overall, 13 participants among the 17 participants were included in the final analysis except for the 4 participants (1 for not completing at least 6 visits and 3 for unclear results of TG and LDL-chol). The results of the paired *t* test comparing before and after treatment indicated statistically significant increases in weight (*P* < .001), height (*P* = .001), and SMM (*P* = .003) with a significant decrease in BMI (*P* = .041) (Table 2). There were no statistically significant differences in the results of blood chemistry before and after treatment (Table 3). The KYPAQ scores decreased from 23.54 ± 2.60 to 22.31 ± 3.82, but were not statistically significant (*P* = .270). In the test of taste sense, preferences for salt density were improved in 8, maintained in 2, and worsened in 3 participants and those for sugar content were improved in 6 and maintained in 7 participants with no worsening. During the entire treatment period, no adverse events were reported.

**Table 2:**
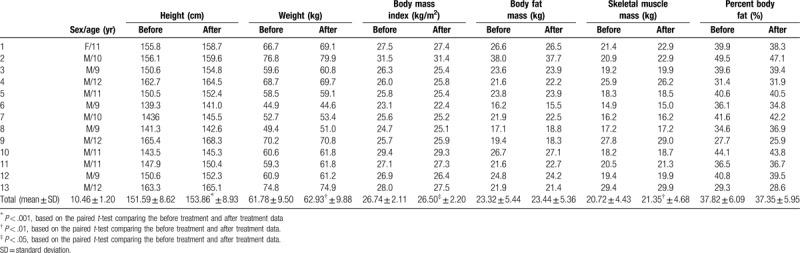
Baseline characteristics and changes in the morphometric parameters.

**Table 3:**
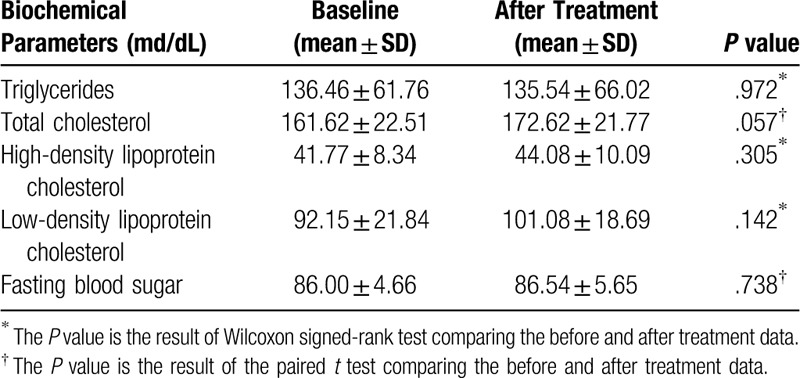
Changes in the biochemical parameters.

## 3 Discussion

Childhood obesity is considered as “one of the most serious public health challenges of the 21st century” by the World Health Organization.^[5]^ As various obesogenic environments contribute to childhood obesity, the socio-ecological model has been used to explain the complex biopsychosocial nature of childhood obesity.^[1]^ Although multi-setting, multicomponent community-based interventions have shown promise in preventing childhood obesity, there is still an inconsistency on the effect of multicomponent community-wide interventions in the available studies.^[15,16]^ Moreover, a few clinical trials have examined the effect of community-based lifestyle modification program combined with herbal medicinal treatment for childhood obesity.^[17]^ In the present study, we showed the clinical effects of an herbal complex, SD, with lifestyle modification in 13 patients with childhood obesity who participated in community-based healthcare program.

In this healthcare program, SD was selected as herbal medicinal treatment among various herbal medicines used in the previous studies on adult or childhood obesity.^[10–14,19,20]^ Herbal medicines, with Ephedra Herba as the main ingredient, have recently been administered as anti-obesity agents based on the results of several experimental and clinical studies.^[10,12,14,17,20]^ However, considering that prescription of herbal medicines, especially for those with strong pharmacological action or with high risk, such as Ephedra Herba, needs to be customized according to individual characteristics to assure the best efficacy and safety,^[14,21]^ SD was adapted in this program because it is more suitable for this generalized prescription process compared to other herbal medicines. SD only consists of herbal medicines with low risk, which are classified as food category and showed anti-obesity effects in the previous experimental studies. SD also showed positive effect on weight loss in a clinical trial with obese premenopausal Korean females.^[19]^

In this study, we showed significantly positive effects of SD with lifestyle modification on BMI, height, and SMM, but with weight gain in patients with childhood obesity, which also suggests its positive effect on one's growth. It is different from the results of the previous studies of Korean medicinal treatments on childhood obesity using herbal medicines usually containing Ephedra Herba, which showed a more positive effect on BMI, weight, and BFM, but none on SMM and height.^[17,20]^ The KYPAQ score used to assess physical activity showed no significant change, which corresponds to the results of a previous systematic review on community-wide interventions for increasing physical activity.^[16]^ In the test for taste sense, the preferences for salt density and sugar content mostly improved or were maintained, which suggests a positive effect on dietary behavior.

Considering that the evaluation of multi-setting, community-based childhood obesity prevention interventions can often be as complex as the problem of obesity itself,^[22]^ the initial purpose of this study, that is, to examine the effect of community-based lifestyle modification program combined with herbal medicinal treatment for childhood obesity, is worth reporting. Moreover, SD showing relatively positive effect only with low risk food category herbs without Ephedra Herba is greatly significant. However, lack of a control group which is a limitation of case series study design and short follow-up period might have led to multiple errors and biases. Further clinical studies with appropriate trial design to confirm the clinical effects of SD are required.

## 4 Conclusion

In this study, we showed significantly positive effects of SD with lifestyle modification on BMI, height, and SMM. The KYPAQ score used to assess physical activity showed no significant change and the preferences for salt density and sugar content mostly improved or were maintained, which suggests a positive effect on dietary behavior. Further clinical studies are needed.

## Acknowledgments

The authors would like to appreciate the contributions of all the participants.

## Author contributions

**Conceptualization:** Koh-Woon Kim, So-Jin Kim, Hojun Kim, Seung-Uoo Shin, Jaechul Song, Won-Seok Chung

**Data curation:** Koh-Woon Kim, Won-Seok Chung

**Formal analysis:** Won-Seok Chung, Hojun Kim

**Investigation:** So-Jin Kim, Seung-Uoo Shin, Jaechul Song

**Methodology:** Koh-Woon Kim, Won-Seok Chung

**Writing – original draft:** Koh-Woon Kim, So-Jin Kim

**Writing – review & editing:** Hojun Kim, Seung-Uoo Shin, Jaechul Song, Won-Seok Chung
